# Transport of Drugs Through Biological Barriers—An Asset or Risk

**DOI:** 10.3390/pharmaceutics17040465

**Published:** 2025-04-03

**Authors:** Anna Weronika Sobańska

**Affiliations:** Department of Analytical Chemistry, Medical University of Lodz, Muszynskiego 1, 90-151 Lodz, Poland; anna.sobanska@umed.lodz.pl

## 1. Introduction

Biological barriers are both cellular and enzymatic interfaces between different compartments of a living organism. Drug molecules administered by the majority of routes must cross one (or more) biological barrier in the body before they reach the site of action [1]. Since the biological barriers protect the respective organs against harmful factors (both physically and enzymatically) and are crucial for the delivery of nutrients and the removal of undesired metabolites, they are relatively complex entities. The passage of a compound across a biological barrier depends on the molecule’s physico-chemical properties, the formulation (if applicable), degree of protein binding, and concentration gradient. Membrane transport may occur via a variety of mechanisms, including the simple or facilitated passive diffusion or active transport [2].

Transport of drugs across biological barriers is an important prerequisite for their activity (although the lack of an intended biological activity does not always imply that the drug cannot penetrate those biological barriers that separate the site of administration from the biological target) [3,4]. On the other hand, drugs’ barrier permeability may also lead to side effects when, for example, a topically active compound applied on the skin surface is absorbed transdermally [5], or a peripherally active drug enters the brain and affects the central nervous system [6]. Similarly, the transport of undesired compounds across biological barriers may occur in the case of environmental contaminants (e.g., pesticides), cosmetic row materials or even textile dyes—a great number of such compounds meet all the possible criteria of drug-likeness in the context of their ability to be absorbed by a human or animal body [7,8].

In this Special Issue, we collected papers covering different aspects of drugs’ and environmental pollutants’ (e.g., pesticides’) transport across biological barriers, the novel in vitro/in silico methods of prediction of barriers’ permeability, and new techniques designed to enhance drugs’ absorption.

## 2. An Overview of Published Articles

In this Special Issue, nine papers were published (listed in chronological order):

(1) Kim, J.; Shin, S.-A.; Lee, C.S.; Chung, H.J. An Improved In Vitro Blood–Brain Barrier Model for the Evaluation of Drug Permeability Using Transwell with Shear Stress. *Pharmaceutics* **2024**, *16*, 48. https://doi.org/10.3390/pharmaceutics16010048.
(2) Lin, G.C.; Friedl, H.-P.; Grabner, S.; Gerhartl, A.; Neuhaus, W. Transport of Non-Steroidal Anti-Inflammatory Drugs across an Oral Mucosa Epithelium In Vitro Model. *Pharmaceutics* **2024**, *16*, 543. https://doi.org/10.3390/pharmaceutics16040543.
(3) Álvarez-Fernández, L.; Blanco-Paniagua, E.; Merino, G. ABCG2 Transports the Flukicide Nitroxynil and Affects Its Biodistribution and Secretion into Milk. *Pharmaceutics* **2024**, *16*, 558. https://doi.org/10.3390/pharmaceutics16040558.
(4) Ren, C.; Ma, Y.; Wang, Y.; Luo, D.; Hong, Y.; Zhang, X.; Mei, H.; Liu, W. Palmitoylethanolamide-Incorporated Elastic Nano-Liposomes for Enhanced Transdermal Delivery and Anti-Inflammation. *Pharmaceutics* **2024**, *16*, 876. https://doi.org/10.3390/pharmaceutics16070876.
(5) Arbitman, L.; Chen, S.; Kim, B.; Lee, M.; Zou, P.; Doughty, B.; Li, Y.; Zhang, T. Assessment of Infant Exposure to Antidepressants through Breastfeeding: A Literature Review of Currently Available Approaches. *Pharmaceutics* **2024**, *16*, 847. https://doi.org/10.3390/pharmaceutics16070847.
(6) Alonso-Cerda, M.J.; García-Soto, M.J.; Miranda-López, A.; Segura-Velázquez, R.; Sánchez-Betancourt, J.I.; González-Ortega, O.; Rosales-Mendoza, S. Layered Double Hydroxides (LDH) as Delivery Vehicles of a Chimeric Protein Carrying Epitopes from the Porcine Reproductive and Respiratory Syndrome Virus. *Pharmaceutics* **2024**, *16*, 841. https://doi.org/10.3390/pharmaceutics16070841.
(7) Basar, E.; Mead, H.; Shum, B.; Rauter, I.; Ay, C.; Skaletz-Rorowski, A.; Brockmeyer, N.H. Biological Barriers for Drug Delivery and Development of Innovative Therapeutic Approaches in HIV, Pancreatic Cancer, and Hemophilia A/B. *Pharmaceutics* **2024**, *16*, 1207. https://doi.org/10.3390/pharmaceutics16091207.
(8) Wanat, K.; Michalak, K.; Brzezińska, E. Log BB Prediction Models Using TLC and HPLC Retention Values as Protein Affinity Data. *Pharmaceutics* **2024**, *16*, 1534. https://doi.org/10.3390/pharmaceutics16121534.
(9) Sobańska, A.W.; Sobański, A.M.; Wanat, K. Pesticides’ Cornea Permeability—How Serious Is This Problem? *Pharmaceutics* **2025**, *17*, 156. https://doi.org/10.3390/pharmaceutics17020156.

Drugs’ release into mother’s milk from the maternal plasma compartment attracted a lot of attention in this SI. Women in lactation are often treated for post-partum depression or other ailments, and due to the lack of data on infants’ exposure to certain drugs through nursing, they are often encouraged to discontinue either their pharmacotherapies, or breastfeeding. Arbitman et al. (5) gave a broad overview of perinatal depression and antidepressant use among breastfeeding females, making the reader more aware of this important issue; they also discussed the factors affecting the milk-to-plasma drug concentration ratio, and presented several in vivo, in vitro, ex vivo, and in silico models used to predict antidepressants’ secretion into breast milk—based on a detailed literature search covering the period from the 1960s to the present day. Álvarez-Fernández, Blanco-Paniagua, and Merino (3) investigated the role of the ABCG2 transporter in the active transport of undesired chemicals into milk. The ABCG2 transporter is present in different barriers (e.g., the blood–brain, the blood–testis and the blood–fetal barrier) and its main task is to remove unwanted compounds from cells. Unfortunately, this protein is also present in the apical membrane of alveolar epithelial cells of the mammary gland, where it facilitates the transport of chemicals into milk. Álvarez-Fernández at al. demonstrated on animal models and using in vitro experiments that the ABCG2 transporter is an important factor enhancing nitroxinil passage into breast milk—and such an enhancement exists in different species, including humans.

Sobańska, Sobański, and Wanat (9) investigated in silico the ability of a large set of pesticides from different chemical families to cross the cornea, and found that the trans-corneal transport of all studied pesticides is possible, although it is more difficult for the majority of highly lipophilic pesticides from the organochlorine and pyrethroid families (the relationship between the logarithmic cornea permeability of compounds and their lipophilicity expressed as logarithm of the octanol–water partition coefficient is approximately reverse-parabolic across a broad lipophilicity range). They concluded also that the studied pesticides, especially those from organochlorine and organophosphorus families, may cause eye corrosion.

Some new developments were made in the understanding of the blood–brain barrier permeability. The structure and functions of this barrier have been studied since the late XIX century [9] and, considering the great significance of brain penetration by chemicals in health and disease, this interest is unlikely to cease. Wanat, Michalak, and Brzezińska (8) proposed new models to predict the ratio of the total drug concentration in the brain to that in blood (log BB) involving drugs’ protein affinity data collected using liquid chromatography on bovine or human serum albumin-modified stationary phases. Kim et al. (1) reported an improved methodology of the blood–brain barrier permeability studies in vitro on immortalized human cells (hCMEC/D3) in trans-wells subjected to shear stress using an orbital shaker. The application of shear stress offers a significant advantage compared to previous models based on similar cell lines, with improved integrity and increased levels of claudin-5 and occludin.

As stated above, an effective delivery of drugs to the site of action is an important prerequisite to their activity, and often a challenge, considering the number of efficient mechanisms protecting living organisms against the exposure to xenobiotics. Basar et al. (7) gave a detailed review of biological barriers that must be overcome by drugs used to treat HIV, pancreatic cancer, and hemophilia A/B. They identified in particular some novel therapeutic approaches (including drug- and disease-specific delivery vehicles and administration routes) designed to enable drugs to cross biological barriers and to reach the site of action. Lin et al. (2) proposed the administration of non-steroidal anti-inflammatory drugs across the buccal mucosa to overcome their adverse effects related to oral administration. The transport of four drugs (celecoxib, diclofenac, ibuprofen, and piroxicam) across the buccal mucosa was studied using an oral mucosa model based on human cell line TR146. The authors proved the suitability of the in vitro oral mucosa model to measure NSAID transport and confirmed the involvement of transporter proteins in the process. Ren et al. (4) proposed a novel methodology to administer palmitoylethanolamide on skin incorporated into elastic nano-liposomes prepared with ceremide, cholesterol, and phosphatidylcholine. They reported an improvement in trans-dermal delivery, anti-nociceptive, and anti-inflammatory effects of encapsulated palmitoylethanolamide compared to a free form. Alonso-Cerda et al. (6) studied the possibility of using layered double hydroxides (LDH’s) as delivery vehicles for vaccines against the porcine reproductive and respiratory syndrome virus. Layered double hydroxides (LDHs) are synthetic, anionic clays with the general formula [M^II^_1−x_M^III^_x_(OH)_2_]^x+^[A^n−^_x/n_/] × yH_2_O (where M^II^—divalent metal cations; M^III^—trivalent metal cations), used as delivery vehicles for several drugs, scaffolds in tissue regeneration, and materials for dental use and vaccine adjuvants, and they were also found to be promising nano-vaccine candidates in terms of stability and immunogenicity in the studied case.

## 3. Conclusions

Since the discovery of the blood–brain barrier in the late 1890s [9] and the first investigations of the influence of the physico-chemical properties of anesthetic drugs on their ability to act in the central nervous system [10], biological barriers have been studied extensively. Mechanisms of compounds’ transport across biological membranes are known in detail and numerous in vivo, ex vivo, in vitro, and in silico models of transport phenomena have been proposed. The Special Issue of *Pharmaceutics* “Transport of Drugs through Biological Barriers—an Asset or Risk” marks approximately the 125th anniversary of Meyer’s memorable publication [10], and the papers presented in this SI cast new light on some interesting aspects of compounds’ passage across biological barriers, in particular the blood–brain barrier, cornea, skin, secretion to breast milk, and buccal mucosa, including valuable reviews of existing knowledge, novel prediction models and promising delivery techniques. We thank all the authors of this Special Issue for their high-quality contributions to such an important area of science and technology.
